# Dexmedetomidine inhibits Tetrodotoxin-resistant Na_v_1.8 sodium channel activity through G_i/o_-dependent pathway in rat dorsal root ganglion neurons

**DOI:** 10.1186/s13041-015-0105-2

**Published:** 2015-03-03

**Authors:** Xi-Yao Gu, Ben-Long Liu, Kai-Kai Zang, Liu Yang, Hua Xu, Hai-Li Pan, Zhi-Qi Zhao, Yu-Qiu Zhang

**Affiliations:** Institute of Neurobiology, Institutes of Brain Science and State Key Laboratory of Medical Neurobiology, Collaborative Innovation Center for Brain Science, Fudan University, 138 Yi Xue Yuan Road, Shanghai, 200032 China; Department of Anesthesiology, Changhai Hospital, The Second Military Medical University, Shanghai, 200433 China; Center for Neuropsychiatric Diseases, Institute of Life Science, Nanchang University, Nanchang, 330031 China

**Keywords:** α2-adrenoceptor, Dexmedetomidine, Dorsal root ganglion, Pain, Tetrodotoxin-resistant (TTX-R) sodium channel Na_v_1.8, Whole-cell recording

## Abstract

**Background:**

Systemically administered dexmedetomidine (DEX), a selective α2 adrenergic receptor (α2-AR) agonists, produces analgesia and sedation. Peripherally restricted α2-AR antagonist could block the analgesic effect of systemic DEX on neuropathic pain, with no effect on sedation, indicating peripheral analgesic effect of DEX. Tetrodotoxin-resistant (TTX-R) sodium channel Na_v_1.8 play important roles in the conduction of nociceptive sensation. Both α2-AR and Nav1.8 are found in small nociceptive DRG neurons. We, therefore, investigated the effects of DEX on the Na_v_1.8 currents in acutely dissociated small-diameter DRG neurons.

**Results:**

Whole-cell patch-clamp recordings demonstrated that DEX concentration-dependently suppressed TTX-R Na_v_1.8 currents in small-diameter lumbar DRG neurons. DEX also shifted the steady-state inactivation curves of Na_v_1.8 in a hyperpolarizing direction and increased the threshold of action potential and decrease electrical and chemical stimuli-evoked firings in small-diameter DRG neurons. The α2-AR antagonist yohimbine or α2_A_-AR antagonist BRL44408 but not α2_B_-AR antagonist imiloxan blocked the inhibition of Na_v_1.8 currents by DEX. Immunohistochemistry results showed that Na_v_1.8 was predominantly expressed in peripherin-positive small-diameter DRG neurons, and some of them were α2_A_-AR-positive ones. Our electrophysiological recordings also demonstrated that DEX-induced inhibition of Na_v_1.8 currents was prevented by intracellular application of G-protein inhibitor GDPβ-s or G_i/o_ proteins inhibitor pertussis toxin (PTX), and bath application of adenylate cyclase (AC) activator forskolin or membrane-permeable cAMP analogue 8-Bromo-cAMP (8-Br-cAMP). PKA inhibitor Rp-cAMP could mimic DEX-induced inhibition of Na_v_1.8 currents.

**Conclusions:**

We established a functional link between α2-AR and Na_v_1.8 in primary sensory neurons utilizing the G_i/o_/AC/cAMP/PKA pathway, which probably mediating peripheral analgesia of DEX.

## Background

Dexmedetomidine (DEX), a potent and highly selective agonist of the alpha 2 adrenergic receptors (α2-ARs) with more favorable pharmacokinetic properties than clonidine (another commonly used α2-AR agonist) is approved for the adult intensive care unit use as sedative infusion by the US Food and Drug Administration in 1999. Three α2-ARs (α2_A_-, α2_B_- and α2_C_-ARs) have been cloned, and all of which are coupled to inhibitory G proteins [1] and play an important role in the control of pain. The α2-ARs have a diffuse distribution in the nervous system, including in primary afferents, spinal dorsal horn and brain stem [2-5]. Systemically administered α2-AR agonists produce anti-nociceptive effects in humans and animals, suggesting that the α2-AR may be involved in anti-nociception at the supraspinal, spinal and peripheral levels [6-9]. Our previous study showed that intrathecal DEX significantly suppressed monoarthritis-induced thermal hyperalgesia and glial activation in spinal level [10]. However, intrathecal or intracerebroventricular administration of DEX produces dose-dependent sedation [11]. Peripherally restricted α2-AR antagonist could block the analgesic effect of systemic DEX on neuropathic pain, with no effect on sedation, indicating peripheral analgesic effect of DEX [7].

Evidence has emerged that the effects on ion channels may be an important mechanism underlying DEX-induced peripheral anti-nociception [12,13]. Previous studies have revealed that changes in function of voltage-gate sodium channels in nociceptive primary sensory neurons participate in the development of peripheral hyperexcitability that occurs in neuropathic and inflammatory pain conditions [14,15]. Among them, the tetrodotoxin-resistant (TTX-R) sodium channel Na_v_1.8 primarily expressed by small- and medium-sized dorsal root ganglion (DRG) neurons [16,17], substantially contributes to the upstroke of action potential in these neurons [18]. Na_v_1.8-null mice displayed a pronounced increase in threshold to noxious mechanical stimuli and a slight decrease in nociceptive thermoreception as well as delayed development of inflammatory hyperalgesia [19]. Likewise, functional knockdown of Na_v_1.8 in rats reduces hyperalgesia and allodynia in neuropathic pain and inflammatory pain models [14,20,21]. Several G-protein-coupled receptors (GPCRs)-mediated second-messenger cascades including PKA, PKC and MAPKs have been shown to regulate Na_v_1.8 sodium channels [14,22]. In the present study, we investigated whether the peripheral DEX-induced analgesia might in part arise from the suppressed activation of TTX-R sodium channel Na_v_1.8 currents via binding to its GPCR α2-ARs in small-diameter DRG neurons.

## Results

### Recording of Nav1.8 currents in DRG neurons

**Figure 1:**
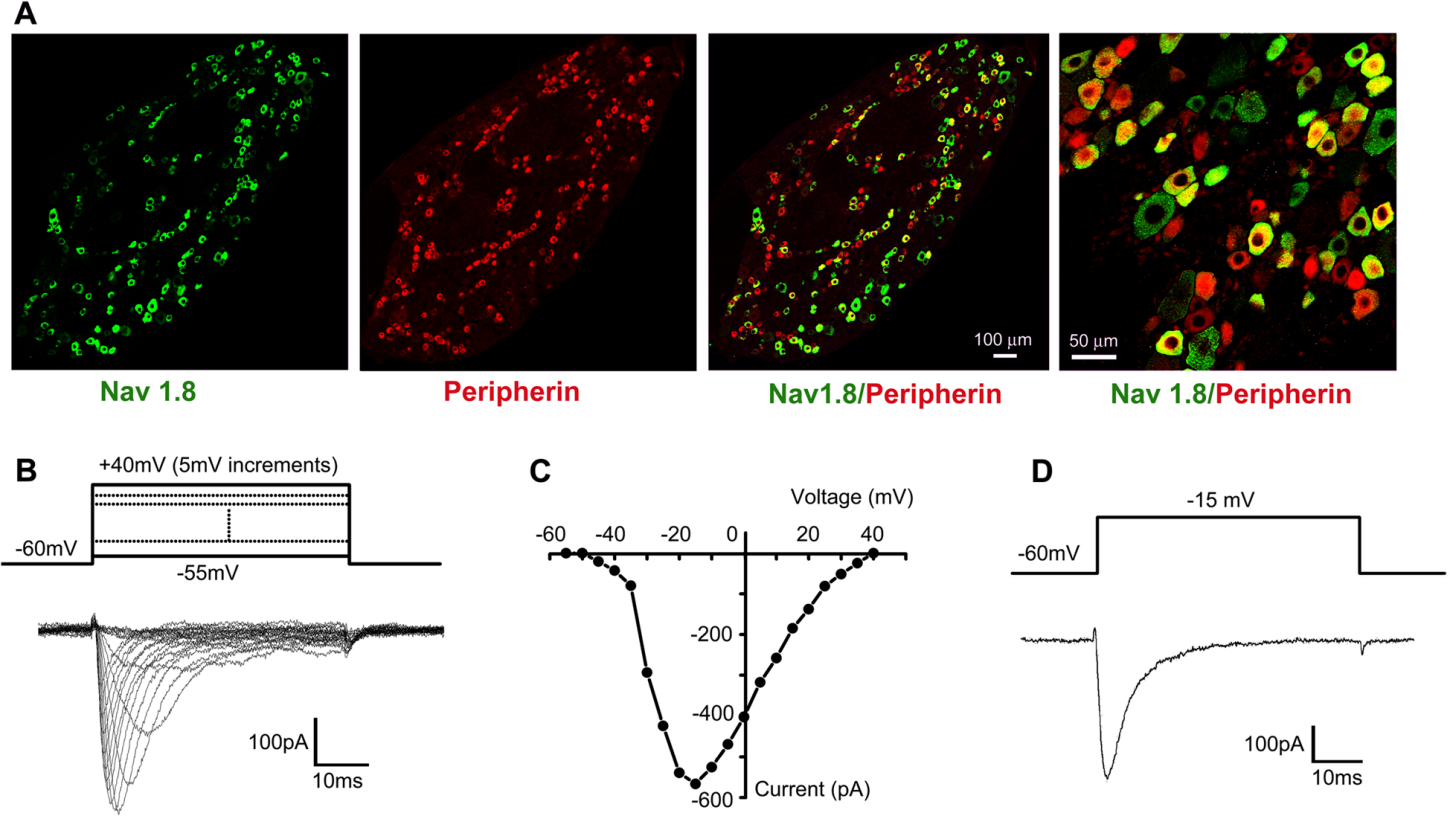
**Isolation of TTX-resistant Na**_**v**_ **1.8 currents in small-diameter DRG neurons. (A)** Double immunofluorescence reveals the expression of Na_v_1.8 in peripherin-positive small-diameter DRG neurons. **(B)** Representative *I-V* curve family of currents recorded in the presence of 500 nM TTX. Cells were depolarized to a variety of potentials (−55 mV to +40 mV) from a holding potential of -60 mV, to elicit Na_v_1.8 currents. **(C)** *I-V* curve of Na_v_1.8 currents shown in (B). **(D)** Representative traces of Na_v_1.8 currents elicited by a single pulse of −15 mV.

Double immunofluorescence revealed that Na_v_1.8 was predominantly expressed in peripherin-positive small-diameter DRG neurons (Figure 1A). In the present study, all recordings were performed in small-diameter (<25 μm) DRG neurons. With existence of TTX (500 nM) in external solution, TTX-resistant (TTX-R) sodium currents were recorded in most (170 out of 223) of the small-diameter DRG neurons. As our previous reported, the membrane potential was held at −60 mV to inhibit Na_v_1.9 currents, leaving the Na_v_1.8 currents intact [22]. The family of Na_v_1.8 currents was generated with a voltage-clamp protocol (holding at -60 mV, depolarizing steps from -55 mV to +40 mV, 50 ms, 5 mV increment, Figure 1B). According to the current–voltage relationship (Figure 1C), we selected -15 mV to elicit Na_v_1.8 currents in most of the recordings (Figure 1D). The peak amplitude of Na_v_1.8 currents was stable during the recordings.

### Effects of DEX on Na_v_1.8 currents in small DRG neurons

**Figure 2:**
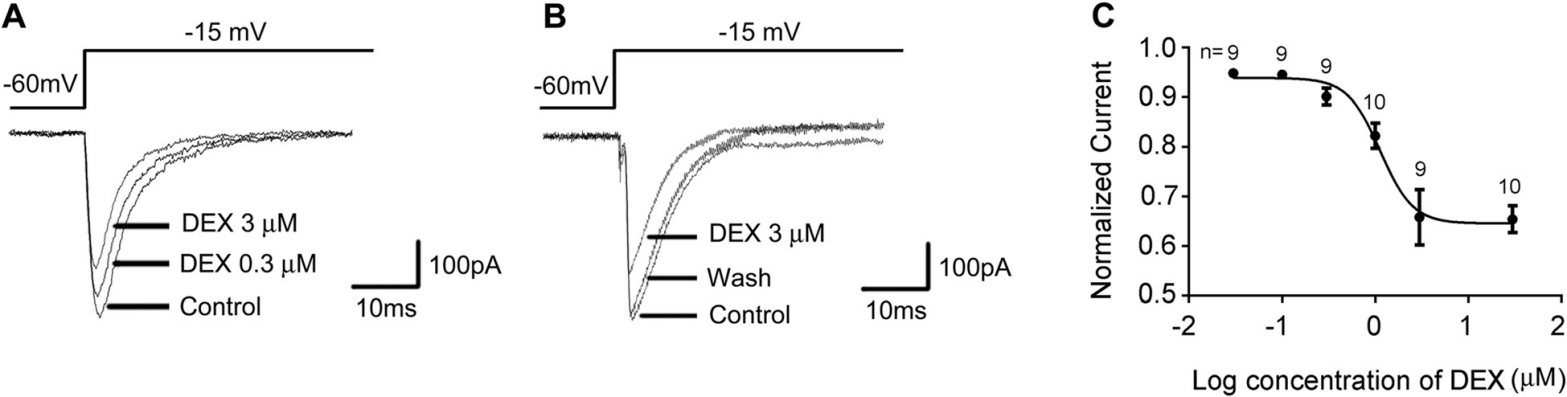
**DEX dose-dependently inhibits Na**_**v**_ **1.8 currents. (A, B)** Typical traces illustrating the Na_v_1.8 currents in small-diameter DRG neurons recorded pre- (control) and post- (DEX) perfusion of DEX and wash out. **(C)** Dose-effect curve of DEX-induced inhibition of Na_v_1.8 currents. The currents were measured after a 1-min application of different concentrations of DEX.

Application of DEX in different doses (0.03, 0.1, 0.3, 1, 3 and 30 μM) dose-dependently reduced the peak amplitude of Na_v_1.8 currents in small DRG neurons within 1 min and washed out within 5 min (Figure 2A and B). One-way ANOVA analysis revealed a significant effect of DEX treatment (F_6,66_ = 23.885, p < 0.001). The ED_50_ was calculated to be 0.92 μM (95% CI: 0.77–1.68). The maximal inhibitory effect (36.51 ± 5.39%) was induced by 3 μM DEX. A higher concentration of DEX (30 μM) failed to induce more powerful inhibition (34.56 ± 2.7%), indicating a “ceiling effect” at a concentration of 3 μM (Figure 2C).

**Figure 3:**
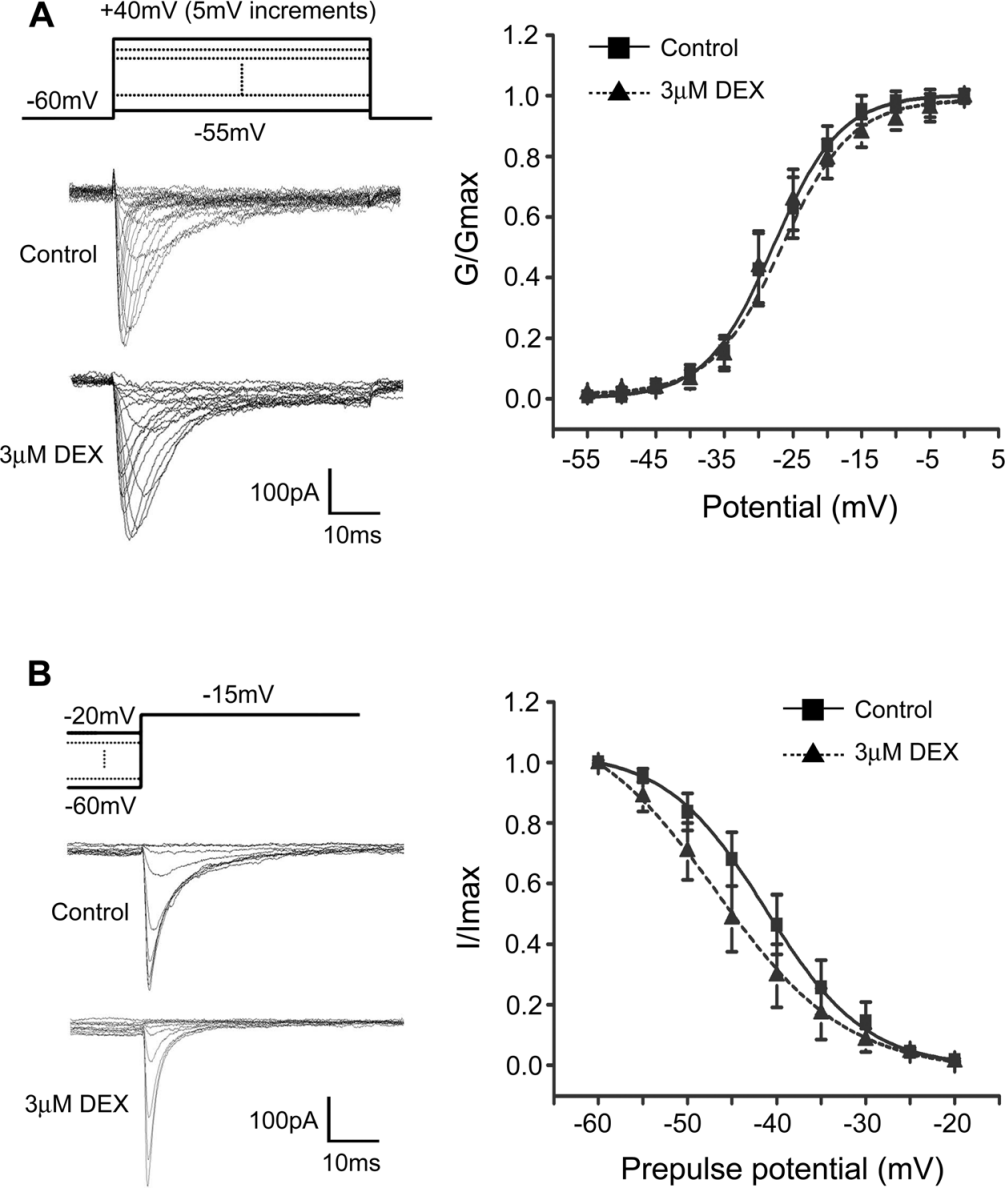
**Effect of DEX on the steady-state activation and inactivation curves of Na**_**v**_ **1.8. (A)** DEX (3 μM) did not shift voltage-dependent activation curve. **(B)** DEX (3 μM) shifted the steady-state inactivation curve in a hyperpolarizing direction.

The effects of DEX on the activation and inactivation properties of Na_v_1.8 currents were studied using the appropriate voltage protocols. As described above, a voltage-clamp protocol consisted of 50 ms depolarizing steps from -55 mV to +40 mV with 5 mV increment was used to determine the activation of Na_v_1.8 channels. No shift in the voltage-dependent activation curve was observed in DEX-treated group compared with control one (Figure 3A). The half-maximal activation potential (V_1/2__activation_) was −27.30 ± 2.13 mV (n = 7) and −28.17 mV ± 0.73 mV (n = 7) in the absence and presence of 3 μM DEX, respectively. Steady-state inactivation of Na_v_1.8 channel was determined at a series of membrane potentials from −60 mV to −20 mV with 5 mV increment for 500 ms and a following test potential of −15 mV. DEX caused a left shift toward the hyperpolarizing potential of the steady-state inactivation curve (Figure 3B). The V_1/2 inactivation_ was −40.49 ± 2.49 mV (n = 9) of the control and −45.39 ± 2.65 mV (n = 9) of DEX treatment, respectively.

### DEX reduced Na_v_1.8 currents via α2_A_-AR

**Figure 4:**
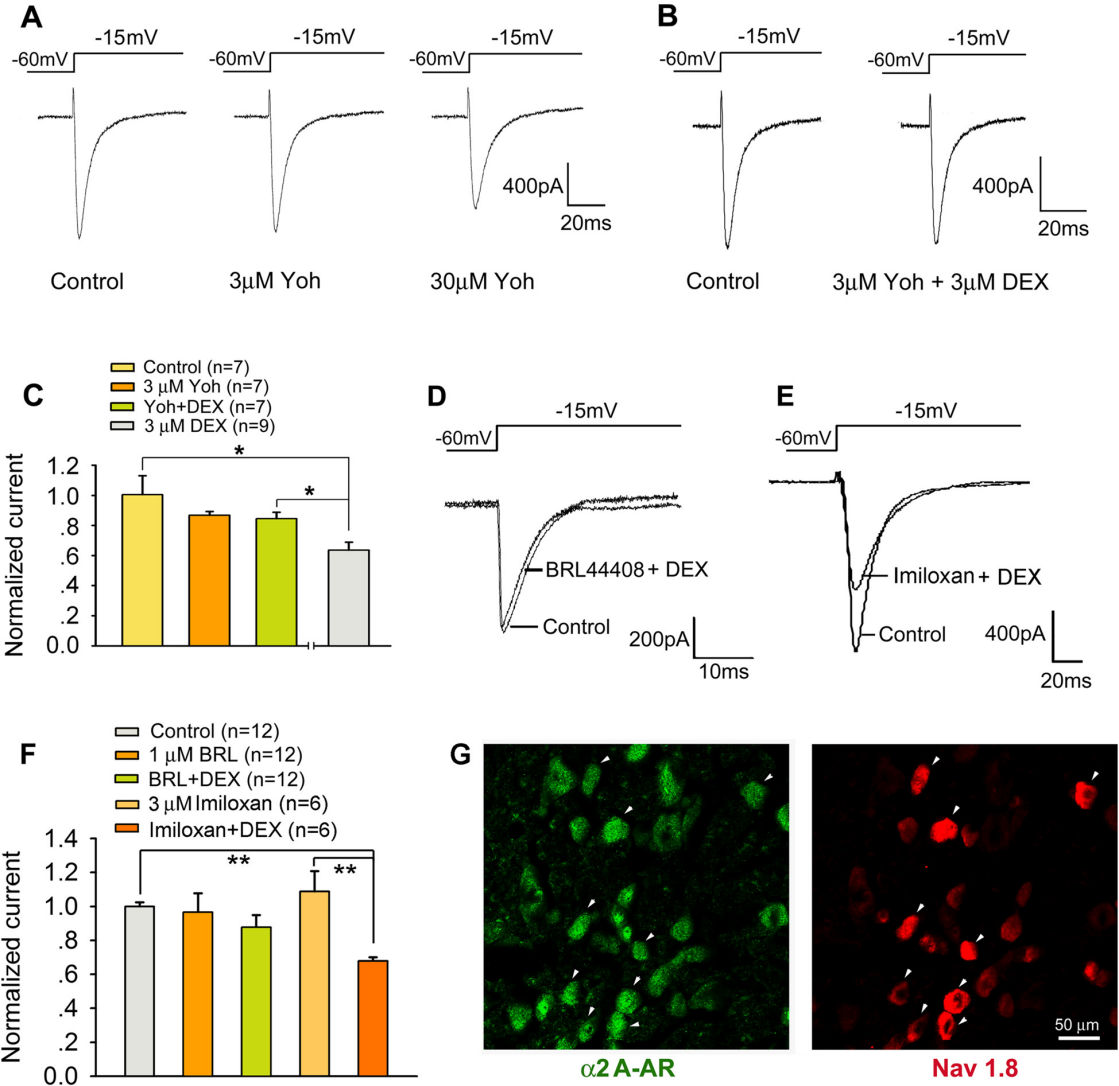
**α2-AR mediates inhibition of DEX on Na**_**v**_ **1.8 currents. (A)** The effects of different doses of yohimbine (α2-AR antagonist) on Na_v_1.8 currents. **(B, C)** pre-incubation of yohimbine (3 μM) for 30 min blocked the inhibitory effect of DEX (3 μM) on Na_v_1.8 currents. *p < 0.05. **(D-F)** DEX-induced inhibition of Na_v_1.8 currents were completely blocked by pre-incubation of α2_A_-AR antagonist BRL44408 (1 μM) but not by α2_B_-AR antagonist imiloxan (3 μM). **(G)** Immunofluorescence micrographs of two adjacent sections from a DRG (L5) after incubation with α2_A_-AR antibody and Na_v_1.8 antibody. Arrowhead indicates coexistence between α2_A_-AR and Na_v_1.8 in the same neurons.

DEX was a selective alpha 2 adrenergic receptor (α2-AR) agonist. To address whether the attenuation of Na_v_1.8 currents induced by DEX application was mediated by α2-ARs, the effect of yohimbine, an α2-ARs antagonist, on inhibitory effects of DEX on Na_v_1.8 currents was examined. Like previous report [12], yohimbine (30 μM) per se inhibited Na_v_1.8 currents (Figure 4A). Pretreatment of DRG neurons with 3 μM yohimbine, a concentration to antagonize DEX-induced membrane hyperpolarization mediated by α2-ARs in rat hypothalamic neurons [23], DEX-induced suppression of the Na_v_1.8 currents was significantly blocked (Figure 4B and C). The peak densities of Na_v_1.8 currents in yohimbine (3 μM) plus DEX (3 μM)-treated group was significantly greater than that in DEX-treated group (One-way ANOVA, F_3, 26_ = 5.451, p < 0.01). Moreover, we examined the effect of BRL44408 (a preferential α2_A_-AR antagonist) on DEX-induced inhibition of Na_v_1.8 currents. Pre-incubation of BRL44408 (1 μM) alone did not affect the peak densities of Na_v_1.8 currents, but significantly blocked DEX-induced suppression of Na_v_1.8 currents (Figure 4D and F). Given that BRL44408 may also be able to block α2_B_-AR at a higher dose, we further examined the effect of α2_B_-AR antagonist imiloxan on DEX-induced inhibition of Na_v_1.8 currents. Neither basal Na_v_1.8 currents nor DEX-induced suppression was influenced by incubation of imiloxan (3 μM) (Figure 4E and F). These data indicated that DEX modulated Na_v_1.8 currents mainly through α2_A_-AR. Also, the colocalization of Na_v_1.8 with α2_A_-AR in DRG small-diameter neurons provided a cellular basis for the involvement of α2_A_-AR in the DEX modulating Na_v_1.8 currents (Figure 4G).

### G_i/o_-proteins participate in DEX-induced Na_v_1.8 currents inhibition

**Figure 5:**
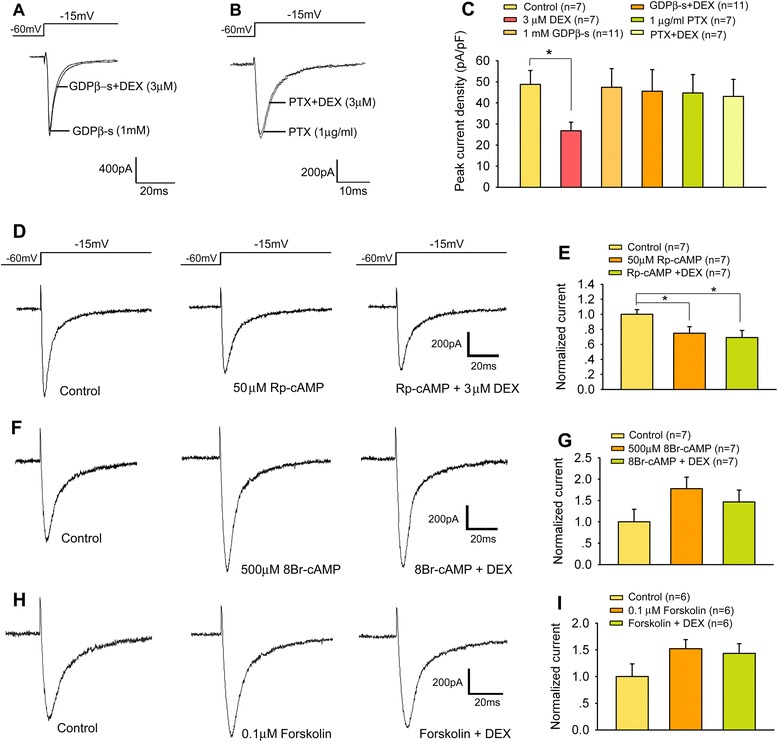
**Involvement of G**_**i/o**_ **proteins/AC/cAMP/PKA pathway in DEX-induced inhibition of Na**_**v**_ **1.8 currents. (A-C)** Loading neurons with 1 mM GDPβ-s or PTX completely abolished the inhibitory effect of DEX on Na_v_1.8 currents. *p < 0.05. **(D, E)** Pre-incubation PKA inhibitor Rp-cAMP (50 μM) inhibited Na_v_1.8 currents. Co-application of DEX did not cause a further reduction of the current amplitudes. *p < 0.05. **(F, G)** Pre-incubation cAMP analogues 8-Br-cAMP (500 μM) prevented DEX-induced inhibition of Na_v_1.8 currents. **(H, I)** Pre-incubation AC activator forskolin (0.1 μM) prevented DEX-induced inhibition of Na_v_1.8 currents.

Given α2-ARs act through G-proteins, we examined the effect of GDPβ-s, a G protein inhibitor, on DEX-induced rapid suppression of Na_v_1.8 currents in small DRG neurons. Inclusion in pipette solution of GDPβ-s (1 mM) did not impair Na_v_1.8 activation. On the other hand, the inhibition of Na_v_1.8 currents by DEX was completely abolished (Figure 5A and C) (One-way ANOVA, F_3,38_ = 12.757, p < 0.01).

α2_A_-ARs are generally known to coupled to the inhibitory G_i_ proteins [24] through which they inhibit adenylate cyclase (AC) activity. Therefore, we examined whether DEX-induced inhibition of Na_v_1.8 currents occurs via Gi proteins. As shown in Figures 5B and C, pertussis toxin (PTX, 1 μg/ml), an irreversible inhibitor of G_i/o_-proteins, significantly prevented the Na_v_1.8 currents amplitude change induced by DEX (One-way ANOVA, F_3,30_ = 15.765, p < 0.01). Inclusion in pipette solution of PTX (1 μg/ml) did not change Na_v_1.8 currents amplitude (Figure 5B and C).

Because G_i_ proteins inhibit the catalytic activity of AC, which catalyzes cAMP production, the G_i_-mediated suppression of Na_v_1.8 currents can be the consequence of decreased levels of intracellular cAMP and a concomitant reduction in PKA-dependent phosphorylation of Na_v_1.8. Here, we used Rp-cAMP, 8-Br-cAMP and forskolin to examine the effects of AC-cAMP-PKA pathway on the Na_v_1.8 currents. Incubate with PKA inhibitor Rp-cAMP (50 μM) for 5 min, Na_v_1.8 currents were significantly suppressed. Co-application of 3 μM DEX did not cause a further reduction of the currents amplitudes (Figure 5D and E). Moreover, 5-min pretreatment of DRG neurons with 8-Br-cAMP (500 μM) a membrane-permeable cAMP analogues, or forskolin (0.1 μM), a AC activator, caused slight increase in Na_v_1.8 currents amplitude (Figure 5 F-I). Although the increase did not reach statistical significance, it totally removed the inhibitory effect of DEX on Na_v_1.8 currents (Figure 5G and I).

### Effect of dexmedetomidine on excitability of DRG neurons

**Figure 6:**
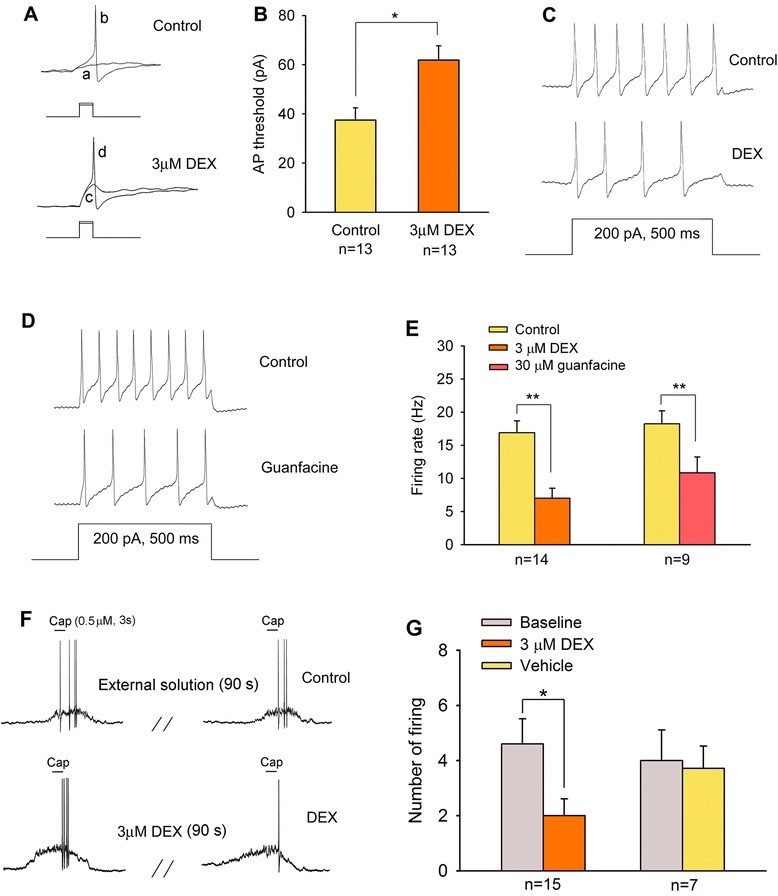
**Effect of DEX on the action potential threshold and firing rate of DRG neurons. (A)** In current clamp model, depolarizing current pulse required to evoke an action potential in a DRG neuron before and after application of DEX (a = 25 pA, b = 30 pA, c = 40 pA, d = 45 pA). **(B)** DEX (3 μM) reduced the amount of currents required to evoke action potential. **(C, D)** Firing response of DRG neurons to a 200 pA depolarizing current pulse (500 ms) before and after application of DEX (C) and selective α2_A_-AR agonist guanfacine (D). **(E)** Summary data indicate the inhibitory effects of DEX and guanfacine on firing rate in DRG neurons. **(F, G)** Current clamp recording showing suppression of capsaicin-induced action potential firing by DEX (3 μM). *p < 0.05; **p < 0.01.

Na_v_1.8 is the main contributor to the upstroke of action potentials in small-diameter DRG neurons [18]. Thus, modulation of this channel by DEX should influence the excitability of DRG neurons. We applied 10 ms step depolarizing currents pulse to evoke action potentials. In 13 of 23 neurons tested, DEX 3 μM significantly increase the injected currents threshold to evoke action potentials from 37.5 ± 4.97 pA to 61.88 ± 5.78 pA before and after exposure to DEX respectively (paired *t*-test, p < 0.01) (Figure 6A and B). Moreover, by injection of maximum currents pulse (500 ms, 200 pA), action potential firing frequencies of DRG neurons were significantly decreased by DEX treatment (Figure 6C and E). DEX-induced inhibitory effect on action potential firing can be mimicked by selective α2_A_-AR agonist guanfacine (30 μM) (Figure 6D and E). Similarly, 0.5 μM capsaicin-induced action potentials were also significantly blocked by DEX (Figure 6F and G).

## Discussion

In this study, we demonstrated that selective α2-AR agonist dexmedetomidine (DEX) reduced Na_v_1.8 currents in small-diameter acutely dissociated DRG neurons. We also showed that DEX decreased excitability of small sensory neurons by increasing the activation threshold and decreasing the action potential firing. This inhibition of Na_v_1.8 currents was completely blocked by the selective α2_A_-AR antagonist, suggesting that α2_A_-AR might be directly involved in DEX-induced changes in Na_v_1.8 activity. We also found that the PTX-sensitive G_i/o_ proteins/AC/cAMP/PKA signaling cascade is primarily responsible for the activation of Na_v_1.8 currents in response to DEX in DRG neurons. These results suggest a peripheral mechanism of DEX analgesia.

TTX-R sodium channel primarily expresses in DRG nociceptors [25,26]. In the two distinct TTX-R sodium channel isoforms Na_v_1.8 and Na_v_1.9, Na_v_1.8 likely mediates the majority of TTX-R currents. Accumulating evidence points up that TTX-R sodium channel plays an important role in peripheral pain processing [27]. Nociceptive signals evoke a dynamic change of TTX-R sodium channel, for example, chronic compression (CCD) of the DRG [15,28] or local inflammation of the DRG by the application of zymosan [29] and subcutaneous injection of carrageenan [30] or complete Freund’s adjuvant (CFA) [31] produces an increase in TTX-R sodium currents in small DRG neurons. Either the physiological or pathological pain was alleviated in the Na_v_1.8-null mice or Na_v_1.8 knockdown rats [14,27,32,33]. Peripheral inflammation or nerve injury has been shown to upregulate Na_v_1.8 expression in nociceptive DRG neurons [34,35]. Blockade of Na_v_1.8 sodium channel by A-803467, a potent and selective Na_v_1.8 sodium channel blocker, could inhibit nerve injury-induced mechanical allodynia and inflammation-induced thermal hyperalgesia [36].

Given that both α2-AR and Nav1.8 are found in small nociceptive DRG neurons [4,5,37], and α2_A_-AR and Na_v_1.8 co-localized in the same small DRG neurons, we propose that stimulation of α2-AR in sensory neurons may lead to an attenuation of the painful symptoms of hypersensitivity via the inhibition of Na_v_1.8 channel activity. Consistently, application of DEX concentration-dependently decreased the current density of Na_v_1.8 in small DRG neurons and shifted the voltage-dependence of steady-state inactivation curve for Na_v_1.8 in the hyperpolarizing direction, which could result in a lower threshold for Na^+^ channel inactivation. DEX also increased the threshold of action potential and decreased firing rate in small DRG neurons. Despite of the previous reports that yohimbine did not alter DEX-induced inhibition of TTX-R Na^+^ currents in small DRG neurons [12] and voltage-gated Na^+^ currents in NG108-15 cells [13], the present study showed that 3 μM yohimbine, a concentration to antagonize DEX-induced membrane hyperpolarization mediated by α2-ARs in rat hypothalamic neurons [23], completed blocked DEX-induced suppression of the Na_v_1.8 currents, suggesting an involvement of α2-ARs in DEX effect. Considering the affinity of yohimbine for α1-ARs, serotonin and dopamine receptors [38], inhibition of high dose yohimbine per se on Na_v_1.8 currents may relate to the interaction of yohimbine with these receptors.

Although three subtypes of the α2-ARs mRNAs were expressed in the rat DRGs [5,39,40], α2_B_-AR mRNA was only found in small numbers of neuron profiles [5,41], and following peripheral nerve injury, α2_A_-AR and α2_C_-AR mRNA levels increased and decreased, respectively [5,40]. Also, the immunohistochemical analysis showed that α2_A_-AR and α2_C_-AR proteins in DRG neurons was respectively increased and decreased after chronic constriction injury of sciatic nerve, whereas no α2_B_-AR neurons were detected in either normal or nerve injury DRG [42]. Moreover, α2_A_-AR rather than α2_C_-AR in the superficial layers of spinal dorsal horn was observed in the terminals of capsaicin-sensitive and substance P-containing primary afferent fibers [2]. Degeneration of TRPV1 afferent terminals, the level of α2_A_-AR, but not α2_C_-AR, was largely reduced in primary afferent terminals [43]. Our present study further showed co-localization of α2_A_-AR- and Na_v_1.8-like immunoreactivity in small DRG neurons. DEX-induced inhibition of Na_v_1.8 currents was prevented by pretreatment of BRL44408, a preferential α2_A_-AR antagonist, but not of imiloxan, a α2_B_-AR antagonist. In addition to Na_v_1.8, other cation channels, for example, TRPM8 may also participate in α2_A_-AR-mediated nociceptive inhibition. Stimulation of α2_A_-AR inhibited TRPM8 in DRG neurons [44]. Taken together, these findings suggest that the α2_A_-AR subtype represents the most likely candidate in DRG neurons to be involved in the modulation of nociceptive information.

Our data strongly suggest that DEX inhibits Na_v_1.8 currents in a G_i/o_ proteins/AC/cAMP/PKA signaling-dependent manner in small DRG neurons. Specifically, we showed that preventing G_i/o_ recruitment by PTX treatment blocked DEX-induced inhibition of Na_v_1.8 current density, and PKA inhibitor mimicked the effect of DEX through the receptor. In support of this, it has been also reported that blockade of PKA activity inhibited the baseline Na_v_1.8 currents in small-diameter nodose ganglion neurons [45]. Consistent with the general notion that stimulation of α2_A_-AR by DEX brings about G_i_-mediated inhibition of AC and reduction of intracellular cAMP levels, AC activator forskolin and cAMP analogues 8Br-cAMP completely reversed DEX-induced inhibition of Na_v_1.8 currents. These findings suggest that the classical AC/cAMP/PKA signaling pathway resulting from the α2-ARs-mediated activation of PTX-sensitive G_i/o_ proteins is involved in the regulation of Na_v_1.8 by DEX.

## Conclusions

DEX attenuated TTX-R sodium channel Na_v_1.8 currents in small-diameter DRG neurons via α2_A_-AR/G_i/o_/AC/cAMP/PKA cascade, which probably constitutes a mechanism of peripheral DEX analgesia.

## Materials and methods

### Animals

Male adult (100–150 g) Wistar rats were obtained from the Experimental Animal Center, Shanghai Medical College of Fudan University, China. Rats were on a 12 h light/dark cycle with a room temperature of 23 ± 1°C and received food and water *ad libitum*. All experiments protocols were permitted by the Shanghai Animal Care and Use Committee and followed the policies issued by the International Association for the Study of Pain on the use of laboratory animals. All efforts were made to minimize animal suffering and reduce the numbers of animals used.

### Preparation of DRG neurons

Animals were anesthetized with ether and rapidly decapitated. DRGs from L4-L6 lumbar segments were removed and immediately transferred onto DMEM (Gibco, Life Technologies, Grand Island, NY, USA) on ice. The ganglia were minced with fine spring scissors and treated with collagenase (2.67 mg/ml, type IA, Sigma, St. Louis, MO) and trypsin (1 mg/ml, type I, Sigma) in DMEM saturated with CO_2_/O_2_ mixed gas at 37°C for 35 min. After wash with standard external solution (in mM, 150 NaCl, 5 KCl, 2 CaCl2, 1 MgCl2, 10 HEPES, and 10 glucose, adjusted to pH 7.4 with NaOH) three times, the ganglia were then gently triturated using fine fired-polished Pasteur pipettes. The dissociated DRG neurons were plated onto 10-mm diameter coverslips in the 3.5 cm culture dishes and incubated with standard external solution. Each culture dishes contained three or four coverslips and all the experiments were performed within 2–8 h after plating.

### Patch-clamp recordings

Whole-cell voltage-clamp and current-clamp recordings of DRG neurons were performed at room temperature (RT, 23 ± 1°C) with an EPC-9 amplifier (HEKA Elektronik, Lambrecht/Pfalz, Germany). Stimulation protocols and data acquisition were controlled by the software Pulsefit 8.5 (HEKA Elektronik). All of the recordings were performed in small-diameter (15–25 μm) DRG neurons with resting membrane potentials more negative than −50 mV. Microelectrodes (N51A borosilicate glass, Sutter Instruments) with a resistance of 2–6 MΩ were pulled using a P97 puller (Sutter Instruments). The pipette solution contained (in mM): 140 CsF, 1MgCl_2_, 1 EGTA, 2.5 Na2ATP, 10 HEPES, pH was adjusted to 7.2 with CsOH. Seals (1–10 GΩ) between the electrode and the cells were established. After the whole-cell configuration was established, the cell membrane capacitance and series resistance were compensated (>80%). Leak currents were subtracted using the online P/4 protocol. The data were sampled at 10 kHz and low-passed at 2 kHz. For Na_v_1.8 recordings, the external solution contained (in mM): 32 NaCl, 20 TEA-Cl, 105 choline-Cl, 1 MgCl_2_, 1 CaCl_2_, 0.1 CdCl_2_, 10 HEPES, 0.0005 TTX and 10 glucose, adjusted to pH 7.4 with NaOH. For current-clamp recordings, the electrode solution was changed to: 140 KCl, 1 MgCl_2_, 0.5 CaCl_2_, 5 EGTA, 10 HEPES, 2.5 Na2ATP, pH was adjusted to 7.2 with KOH. The external solution was changed to: 150 NaCl, 5 KCl, 2.5 CaCl_2_, 1 MgCl_2_, 10 HEPES, pH was adjusted to 7.4 with NaOH. DRG neurons were held at −60 mV and Na_v_1.8 currents were evoked by depolarizing pulses to -15 mV. The activation and inactivation properties of Na_v_1.8 currents were studied using the appropriate voltage protocols. The voltage-clamp protocol consisted of 50 ms depolarizing steps from -55 mV to +40 mV with 5 mV increment was used to determine the activation of Na_v_1.8 channels. The Boltzmann function of the form *G*_*Na*_*/ G*_*Namax*_ *= 1 / {1 + exp [(V*_*m1/2*_*- V*_*m*_*) / k]}* was used to describe the voltage dependence of activation and half activation potential was obtained. Steady-state inactivation of Na_v_1.8 channel was determined at a series of membrane potentials from −60 mV to −20 mV with 5 mV increment for 500 ms and a following test potential of −15 mV. The steady-state inactivation curve was fitted by the Boltzmann function *I*_*Na*_*/ I*_*Namax*_ *= 1 / (1 + exp [(V - V*_*m1/2*_*) / k])*, where *I*_*Namax*_ is the maximal peak current, *V* is the prepulse membrane potential.

### Drugs

All the drugs were purchased from Sigma (St. Louis, MO, USA). The drugs were dissolved in normal saline as stock solutions. All of the stock solutions were stored at −20°C or −80°C until use. Working concentrations of the drugs were prepared on the day of the experiment from the stock solutions. The drug dosages were selected based on previous reports and our preliminary studies. Dexmedetomidine was applied continuously for 1 min closed to cells via ALA-VM8 perfusion system (ALA Scientific Instruments, Westbury, NY). Yohimbine and BRL44408 were applied to the chamber 30 min before and during the perfusion of dexmedetomidine (DEX). Rp-cAMP, 8-Br-cAMP and forskolin were applied to chamber 5–10 min before and during the DEX perfusion. GDPβ-s and PTX were applied in the pipette internal solution.

### Immunohistochemistry

Animals were given an overdose of urethane and were then transcardially perfused with normal saline followed by 4% paraformaldehyde in 0.1 M phosphate buffer (pH 7.4, 4°C). DRGs (L4–L6 segments) were removed and postfixed in the same fixative for 2 h at 4°C and then immersed in a 10–30% gradient of sucrose in phosphate buffer for 24–48 h at 4°C for cryoprotection. DRGs were embedded in OCT compound, cut in a cryostat (Leica 1900, Leica) at 7 μm (to study Na_v_1.8 and α2_A_-AR coexistence) or 14 μm thickness and mounted onto gelatin coated slides. The sections were placed in a humid chamber and processed for immunohistochemistry. The sections were blocked with 10% donkey serum in 0.01 M PBS (pH 7.4) with 0.3% Triton X-100 for 1 h at RT. For Na_v_1.8 and peripherin (a small-diameter DRG neuronal marker) double immunofluorescence, the sections were incubated with a mixture of rabbit anti-Na_v_1.8 (1:1000; Alomone) with mouse anti-peripherin (1:2000; Millipore) overnight at 4°C, followed by a mixture of Alex Flour 488- and Alex Flour 546-conjugated secondary antibodies (1:200; Invitrogen) for 2 h at 4°C. For detecting Na_v_1.8 and α2_A_-AR coexistence, two adjacent sections (7 μm) was respectively incubated with rabbit anti-Na_v_1.8 (1:1000) and rabbit anti-α2_A_-AR (1:100; Alomone) primary antibodies in PBS with 1% normal donkey serum and 0.3% Triton X-100 overnight at 4°C, followed by incubation within Alex Flour 546- and Alex Flour 488-conjugated secondary antibodies for 2 h at 4°C, respectively. All of the slides were coverslipped with 50% glycerin in 0.01 M PBS and then examined with an Olympus FV1000 confocal laser scanning microscope (Olympus). Images were acquired using FV10-ASW software. The specificities of the immunostaining were verified by observing no immunostaining after omitting the primary antibodies, which resulted in the disappearance of the immunostaining signals. The specificities of the primary antibodies were verified by a preabsorption experiment. Sections were first incubated overnight with a mixture of Na_v_1.8 or α2_A_-AR primary antibody and the corresponding blocking peptide (5:1 blocking peptide: primary antibody), followed by incubation with a secondary antibody. The immunostaining signals were abolished after absorption.

### Data analysis

The data were presented as means ± standard error of mean (SEM). Statistical comparisons were performed using Student’s *t*-test, paired *t*-test and one-way ANOVA followed by *post hoc* Student-Newman-Keuls test. In all cases, p < 0.05 was considered statistically significant.
